# Visualising spatial heterogeneity in glioblastoma using imaging habitats

**DOI:** 10.3389/fonc.2022.1037896

**Published:** 2022-11-24

**Authors:** Mueez Waqar, Petra J. Van Houdt, Eline Hessen, Ka-Loh Li, Xiaoping Zhu, Alan Jackson, Mudassar Iqbal, James O’Connor, Ibrahim Djoukhadar, Uulke A. van der Heide, David J. Coope, Gerben R. Borst

**Affiliations:** ^1^ Department of Neurosurgery, Geoffrey Jefferson Brain Research Centre, Manchester Centre for Clinical Neurosciences, Northern Care Alliance NHS Foundation Trust, Manchester Academic Health Sciences Centre, Manchester, United Kingdom; ^2^ Division of Cancer Sciences, School of Medical Sciences, Faculty of Biology, Medicine and Health and Manchester Cancer Research Centre, University of Manchester, Manchester, United Kingdom; ^3^ Department of Radiation Oncology, the Netherlands Cancer Institute, Amsterdam, Netherlands; ^4^ Department of Neuroradiology, Geoffrey Jefferson Brain Research Centre, Manchester Centre for Clinical Neurosciences, Northern Care Alliance NHS Foundation Trust, Manchester Academic Health Sciences Centre, Manchester, United Kingdom; ^5^ Division of Informatics, Imaging and Data Sciences, Faculty of Biology, Medicine and Health and Manchester Cancer Research Centre, University of Manchester, Manchester, United Kingdom; ^6^ Department of Radiology, The Christie NHS Foundation Trust, Manchester, United Kingdom; ^7^ Department of Clinical Oncology, The Christie NHS Foundation Trust, Manchester, United Kingdom

**Keywords:** glioblastoma, imaging, biomarker, habitats, MRI, preoperative, heterogeneity

## Abstract

Glioblastoma is a high-grade aggressive neoplasm characterised by significant intra-tumoral spatial heterogeneity. Personalising therapy for this tumour requires non-invasive tools to visualise its heterogeneity to monitor treatment response on a regional level. To date, efforts to characterise glioblastoma’s imaging features and heterogeneity have focussed on individual imaging biomarkers, or high-throughput radiomic approaches that consider a vast number of imaging variables across the tumour as a whole. Habitat imaging is a novel approach to cancer imaging that identifies tumour regions or ‘habitats’ based on shared imaging characteristics, usually defined using multiple imaging biomarkers. Habitat imaging reflects the evolution of imaging biomarkers and offers spatially preserved assessment of tumour physiological processes such perfusion and cellularity. This allows for regional assessment of treatment response to facilitate personalised therapy. In this review, we explore different methodologies to derive imaging habitats in glioblastoma, strategies to overcome its technical challenges, contrast experiences to other cancers, and describe potential clinical applications.

## Introduction

Glioblastoma is the most common form of primary brain cancer with a median survival of just 15 months (1). The treatment outcome of this tumour has not changed in decades and there are increasing efforts to personalize care for glioblastoma patients. This includes novel strategies that deliver intensified upfront treatment around the time of diagnosis, such as preoperatively, to prevent the phenomenon of rapid early progression, a strongly negative prognostic factor (2, 3). These approaches could improve the treatment outcome and require robust non-invasive tools to monitor treatment response. For glioblastoma, this should be on a regional basis given its significant spatial heterogeneity (4).

Intra-tumoral spatial heterogeneity is a well-recognised phenomena in glioblastoma, especially at the genomic and transcriptomic levels (5, 6). Studies utilising multiple regional sampling have described spatially distinct expression of key driver mutations including Epithelial Growth Factor Receptor (EGFR), TP53 and neurofibromatosis type 1 (NF1), and also the presence of at least two transcriptomic Verhaak classes within the same tumour in up to 60% of cases (5). At the microscopic level, spatial heterogeneity can also be appreciated by the presence of distinct tumour niches, which are groups of cells localising to particular regions within the tumour microenvironment. The perivascular niche for example, includes endothelial cells in close proximity to glioblastoma cancer stem cells (7). Tumour niches are characterised by distinct gene expression patterns that could influence response to treatment (8). On a macroscopic level, there is currently no robust method to detect glioblastoma’s spatial heterogeneity, which could otherwise aid patient stratification for early time-point clinical trials for example.

**Figure 1:**
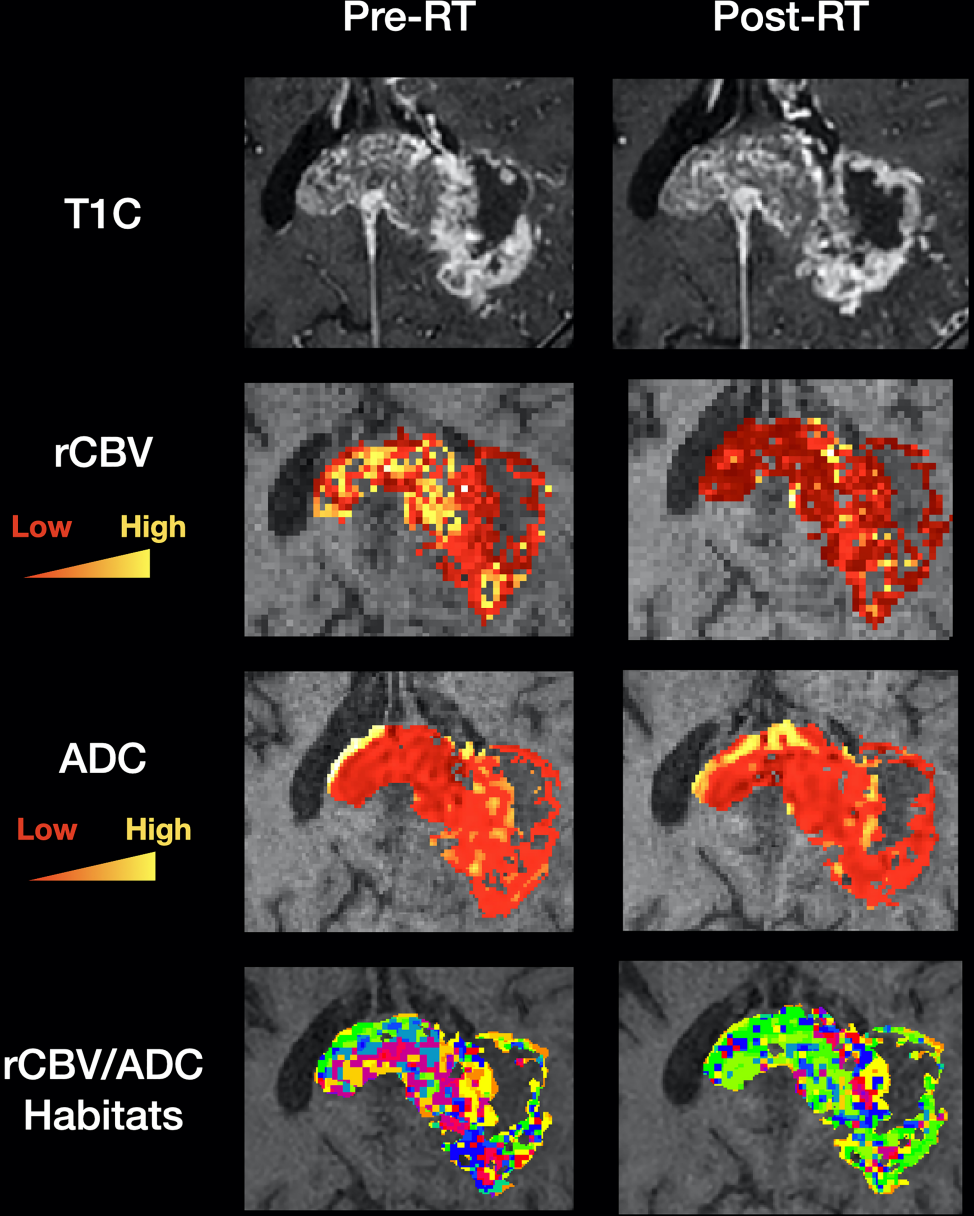
Clinical utility of habitat imaging in glioblastoma: assessment of changes pre and post-radiotherapy. This figure demonstrates the clinical utility of habitat imaging in glioblastoma pre and post-radiotherapy. Top row – structural imaging (T1 with contrast) demonstrates no significant changes in tumour anatomy. Middle two rows – diffusion and perfusion MRI scans demonstrate changes in tumour physiology with treatment with a decrease in rCBV for example (red to yellow represents low to high values for each biomarker). Bottom row – imaging habitats map where each voxel is labelled according to both rCBV and ADC values. This method produced 16 different habitats for this patient. After radiotherapy, the biggest increase was in a habitat defined by low rCBV and low ADC (10.5% increase). The biggest decrease was in a habitat defined by high rCBV and medium ADC (5.7% decrease). Habitats that are more resistant to treatment can be spatially visualised and offered targeted therapy. RT, radiotherapy; T1C, T1 with contrast; rCBV, relative cerebral blood volume normalised to contralateral white matter; ADC, apparent diffusion coefficient.

Magnetic resonance imaging (MRI) is used to guide glioblastoma treatment including surgery and radiotherapy, and could be used to monitor treatment response on a regional basis. To date, most efforts utilising MRI data in glioblastoma have focussed on radiomic approaches to extract innumerable quantitative imaging metrics with less emphasis on spatially relating these to the tumour microenvironment. Habitat imaging is an emerging imaging technique to delineate the tumour into distinct spatial regions with shared imaging characteristics. These regions can be visualised and interrogated longitudinally to characterise tumour regions and monitor their treatment response (**Figure 1**).

Traditional approaches to delineating imaging habitats in glioblastoma have considered regions based on their location on structural MRI sequences (optimised for visualising brain anatomy). For example, at least five habitats could be defined by considering just two structural imaging sequences – T1 with gadolinium and Fluid Attenuated Inversion Recovery (FLAIR): the necrotic core, peri-necrotic enhancing rim, enhancing core, enhancing rim and the ‘infiltrative zone’ defined by FLAIR hyperintensity in the absence of contrast enhancement (9, 10). However, these regions are not always easy to segment and their size is defined by arbitrary and subjective thresholds. The regions themselves are also inherently heterogeneous – for example, the non-enhancing FLAIR hyperintensity is a mixture of oedema and infiltrative tumour with no clear delineation between them. There is therefore a need for alternative methods of deriving imaging habitats in glioblastoma.

In this review, we will provide an overview of the current status of habitat imaging in glioblastoma, highlighting its potential use as a non-invasive tool for more personalised treatment. We will explore different methodologies to derive imaging habitats, strategies to overcome its technical challenges, contrast experiences to other cancers, and describe potential clinical applications.

## Imaging biomarkers

A biomarker is defined as a characteristic that is measured as an indicator of normal biological processes, pathogenic processes or responses to an exposure or intervention, including therapeutic interventions (11). Imaging biomarkers are biomarkers that are derived from clinical imaging sequences such as MRI. Examples of conventional imaging biomarkers used in glioblastoma derived from diffusion and perfusion MRI are listed in **Table 1**.

**Table 1:** Conventional imaging biomarkers in glioblastoma (12).

Imaging biomarker	Details
Apparent diffusion coefficient (ADC)	Measurement of inferred (‘apparent’ rather than actual) water diffusion with DWI. It is a measure of the relative decrease in the transverse magnetization induced by additional dephasing and rephasing magnetic field gradients. Net dephasing and therefore signal loss is greater in freely diffusive tissue. Quantitatively, the ADC is the slop of a line plotting the natural logarithm of the MRI signal (y-axis) per unit of applied magnetic field strength (b-value plotted on x-axis; units mm^2^/s).
Mean diffusivity (MD)	This is the magnitude of mean diffusion in a given voxel obtained with diffusion tensor imaging (DTI). ADC may not be uniform at all orientations. MD is the average diffusivity from the three eigenvalues of the diffusion tensor. It is often regarded as an approximation of the overall ADC (units mm^2^/s).
Fractional anisotropy (FA)	DTI provides FA values which indicate the overall directionality of water diffusion within a voxel. FA is a scalar value between 0-1 that describes the degree of anisotropy of the diffusion process. A value of zero means that diffusion is isotropic (i.e. equal in all directions, and the diffusion ellipsoid is a sphere). A value of one means that diffusion is totally anisotropic (i.e. diffusion occurs only along one axis and is fully restricted along all other directions).
Cerebral blood volume (CBV)	CBV is the volume of blood in a given amount of brain tissue, most commonly millilitres of blood per 100 g of brain tissue. CBV can be calculated by assessing the area under the concentration-time curve, which in turn can be generated from signal intensity-time curves generated using Dynamic Contrast Enhanced (DCE) MRI (measuring T1 signal recovery) or Dynamic Susceptibility Contrast (DSC) MRI (measuring T2 signal loss), respectively (units ml/100g).
Cerebral blood flow (CBF)	Cerebral blood flow is the volume of blood passing through a given amount of brain tissue per unit of time, most commonly millilitres of blood per minute per 100 g of brain tissue. Alternatively, one may express CBF in terms of flow per unit volume of brain tissue, thus in ml blood/min/100 ml tissue.
Mean transit time (MTT)	Mean transit time is the average period of time that blood spends within the blood vessels in a particular part of the brain (units seconds).
Volume transfer constant (*K* ^trans^)	*K* ^trans^ is the volume transfer constant for contrast agent between blood plasma and the tissue extravascular extracellular space (EES). *K* ^trans^ is derived from a pharmacokinetic model and represents a mix of flow and permeability. It most commonly serves as a measure of permeability/vascular leak under permeability-limited conditions (units min^-1^).
Rate constant (*k*_ep_)	*k*_ep_ determines the washout rate of contrast agent from the extravascular extracellular space back into the blood plasma (k_ep_ = K^trans^/v_e_; units min^-1^).
Extravascular extracellular space fractional volume (*v_e_*)	*v*_e_ is defined as the volume of the extravascular extracellular space (EES) per unit volume of tissue, and thus is a dimensionless number between 0 and 1. The parameter *v*_e_ reflects the amount of “room” available within the tissue interstitium for accumulating contrast agent. Note that *v*_e_ is different from *V*_e,_ which represents the total volume of extravascular extracellular space in ml.
Fractional plasma volume (*v_p_*)	Represents the volume of blood plasma per unit volume of tissue (therefore unitless). It is derived from a pharmacokinetic model.
Native longitudinal relaxation rate (R1_N_)	R1 is the longitudinal relaxation rate of the protons of tissue water (R1 = 1/T1). R1_N_ is the baseline tissue R1 in the absence of the contrast agent. The R1_N_ measurement inversely reflects the free water content of tissue (units s^-1^).

This table provides an overview of the most commonly cited imaging biomarkers used in glioblastoma patients. Note that the prefix of ‘r’ before these imaging biomarkers represents comparison to a reference region, that is usually the contralateral normal appearing brain parenchyma, but defined differently from study to study.

In addition to conventional imaging biomarkers, it is possible to apply data-mining approaches to imaging data to yield quantifiable data, under the theme of radiomics. Radiomics typically produces a vast set of imaging features that are derived from the tumour as a whole. This feature set is a distinct imaging biomarker in its own right that is useful for aiding in diagnosis, prognostication and predicting treatment response (13). Although this may have advantages to histopathological analysis by decreasing the likelihood of intraoperative under-sampling by considering the tumour as a whole (14), it does not typically relate imaging metrics to individual tumour regions. Tumour subregion radiomic analyses have also focused on relating radiomic features to patient related outcomes, relaying little about the underlying tumour microenvironment limiting its use in guiding novel treatment strategies (15). This limitation of assessing regional response may be overcome by enhanced use of conventional imaging biomarkers used in isolation/together.

Imaging biomarkers provide information about tumour biological characteristics with varying specificity. In current practice, imaging biomarkers are largely used in isolation, which is advantageous given the simplicity of this approach. However, there may be benefit in combining different biomarkers using the additional and differential information provided by considering their overlapping areas. In one study for example, the positive predictive value (PPV) of relative cerebral blood volume (rCBV; defined in **Table 1**), apparent diffusion coefficient (ADC; defined in **Table 1**) and the FLAIR signal, to predict disease recurrence in glioblastoma was evaluated. The PPV for recurrence was improved by considering the overlap of high FLAIR, rCBV and low ADC (PPV = 31.9%), versus individual biomarkers alone (PPV for rCBV = 21.6%) (16).

Habitat imaging utilises imaging biomarkers to delineate distinct spatial regions with homogenous biological and physical characteristics within an individual tumour (17). This has specific applications in glioblastoma as identification of more aggressive/treatment resistant habitats could enable locally targeted treatment, such as targeted resection for hypoxic areas for example, that correlate with a shorter survival (18). Habitat imaging could also overcome limitations in the sensitivity of individual imaging biomarkers in assessing and monitoring multiple physiological processes, and provide a more accurate representation of the tumour molecular profile non-invasively (19, 20).

## Habitat imaging definition

Cancer exhibits marked spatial heterogeneity at the anatomical, physiological and molecular levels (21). Imaging can be interrogated to visualize this spatial heterogeneity and identify imaging habitats (17). Imaging habitats are tumour regions with distinct imaging characteristics that arise from their unique intrinsic cell populations and/or local environmental conditions. Although individual imaging biomarkers could be used in theory to define habitats, based on analysis of voxel signal intensity distributions for example, it is more conventional to use the term when tumour regions are defined using multiple imaging biomarkers. Thus, for the purposes of this review, imaging habitats will refer to tumour regions defined using multiple imaging biomarkers. As each imaging biomarker assesses a different aspect of tumour biology, a multiple biomarker approach also increases the degree of tissue heterogeneity that can be assessed.

## Habitat imaging in glioblastoma: Status and potential

### Current experience

**Figure 2:**
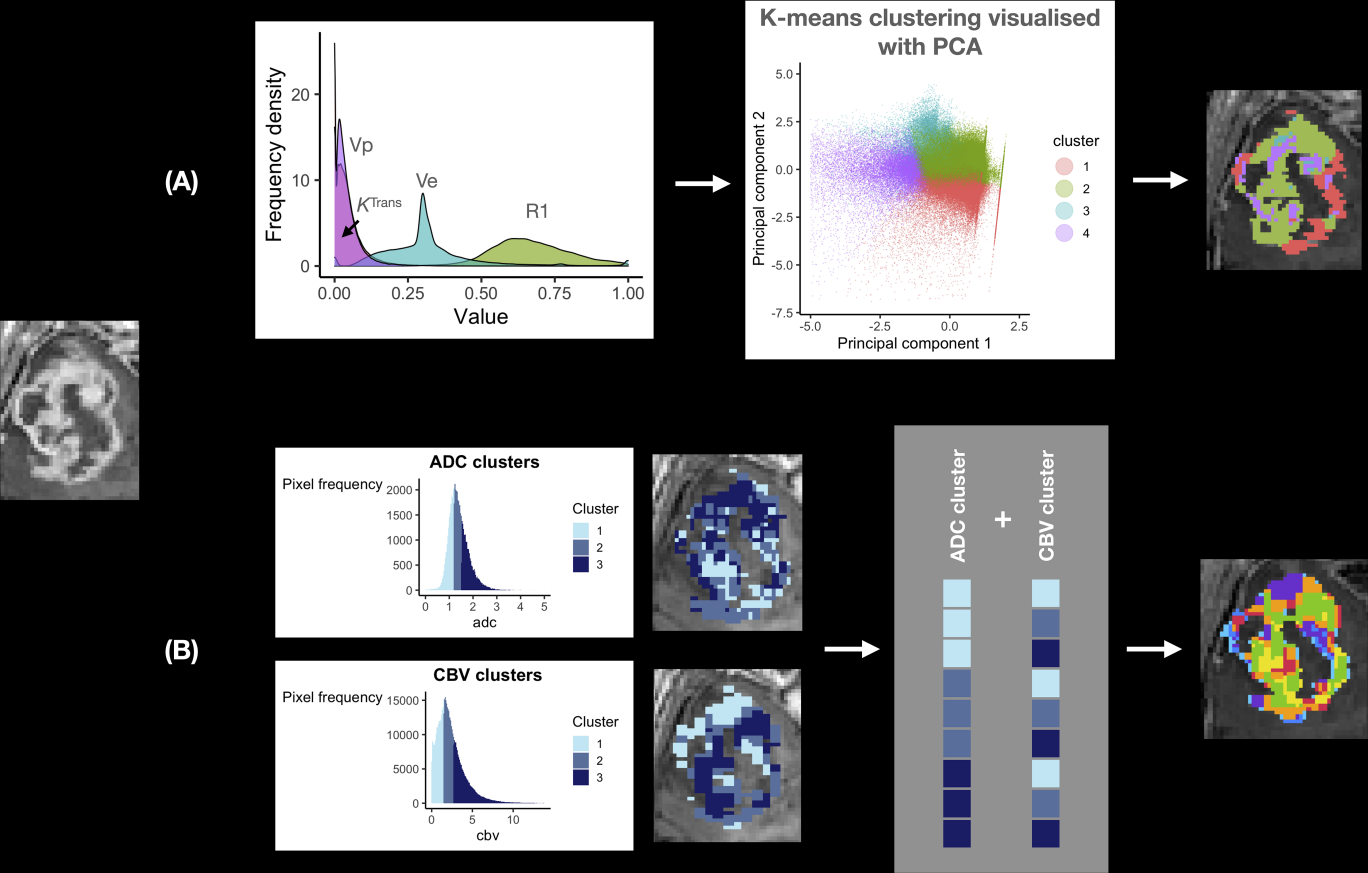
Habitat imaging methods in glioblastoma. This figure provides an overview of the two main approaches to deriving imaging habitats utilising local preoperative data from 12 patients with glioblastoma undergoing surgery. **(A)** one step approach: a multi-dimensional dataset can be produced utilising multiple imaging biomarkers from the same MRI acquisition (to avoid interpolation/registration errors), in this case Dynamic Contrast Enhanced (DCE) MRI. Data from R1_N_ – defined in **Table 1** and three DCE-MRI imaging biomarkers (*K* ^trans^, *v_p_* and *v_e_*) were input into a machine learning K-means clustering algorithm to produce four distinct imaging habitats, that were distinct on Principal Component Analysis (PCA; right). A disadvantage of this approach is its ‘black-box’ nature, such that it is not straightforward to define each habitat for prospective validation. **(B)** Two step approach: this step first requires clustering of individual imaging biomarkers, in this case ADC and rCBV (left). Each pixel is then assigned to a habitat based on its ADC/rCBV cluster, with multiple cluster combinations defining each habitat (grey box). The advantage of this approach is that imaging biomarkers from different MRI acquisitions (e.g. diffusion and perfusion MRI) can be utilised. It is also easier to define each habitat as the definition of each is derived from its individual ADC/rCBV cluster composition. This approach therefore allows for prospective validation with pre-defined cluster thresholds.

Several studies have investigated the potential of habitat imaging for predicting relevant clinical endpoints in glioblastoma (**Table 2**). **Supplementary Figure 1** outlines the search strategy and methodology used for this section. In general, there are two main approaches to habitat imaging (**Figure 2**). The first (‘one step’) involves using bioinformatics to cluster multi-dimensional imaging biomarker datasets. In this approach, data from multiple imaging biomarkers is combined into a common data table and clustering methods such as hierarchical clustering are used to identify groups (27, 30). The second approach involves two steps, in which data from each individual biomarker is firstly split into data clusters and multiple combinations of those clusters can be used to define habitats (26).

**Table 2:** Habitat imaging in glioblastoma: the current evidence.

Paper	Patients	Imaging habitats							Survival/progression	Histopathology	Molecular
		Stage of imaging	MRI sequences/biomarkers	Intensity normalisation	Biomarker clustering		Habitat generation				
Lee 2015 Texas, USA (22)	Glioblastoma N = 74 from The Cancer Genome Atlas	Preoperative	T1+C FLAIR	Yes	Gaussian mixture modelling - 2 clusters		Overlap of each cluster to produce 4 habitats		Top 5 spatial features had good area under curve = 0.76 for predicting survival	Top 5 spatial features had high accuracy for predicting subtypes - pro-neural 0.93, classical 0.88, neural 0.85, mesenchymal 0.70	
Lee 2015 Texas, USA (23)	Glioblastoma N = 65 from The Cancer Genome Atlas	Preoperative	T1+C FLAIR	Yes	As above						Top 5 spatial features had high accuracy for predicting EGFR status (AUC 0.845)
McGarry 2016 Single-centre Wisconsin, USA (24)	Glioblastoma N = 81	Preoperative	T1 T1+C FLAIR ADC	Yes	Automated tissue segmentation – 3 clusters		Overlap of each cluster to produce 83 habitats		Identified 5 habitats associated with shorter overall survival High intensity of both FLAIR and contrast enhancement present in 4 out of 5 habitats associated with overall survival	One habitat could be hyper-cellular	
Zhou 2016 Florida (25)	Glioblastoma N = 32 from The Cancer Genome Atlas N = 22 internal cohort	Preoperative	T1+C T2 FLAIR	Yes	Otsu thresholding – 2 clusters		Interested in two habitats formed by overlap of high/high or low/low clusters		Spatial mapping between habitats were better at predicting survival than the presence of habitats themselves In particular, spatial mapping between high T2/high FLAIR region achieved >80% accuracy		
Khalifa 2016 Toulouse, France (16)	Glioblastoma N = 15 Primary glioblastoma enrolled in a trial, ≤5cm diameter	Postoperative pre-radiotherapy	FLAIR rCBV ADC	NR	Gaussian mixture modelling to produce 2 (ADC) and 3 (rCBV) clusters		Interested in 4 habitats formed by various overlap combinations		Positive predictive value for recurrence was highest at 31.9% in overlap region of high FLAIR, hyper-perfusion and restricted diffusion		
Dextraze 2017 Texas, USA (26)	Glioblastoma N = 85 from The Cancer Genome Atlas	Preoperative	T1 T1+C T2 FLAIR	Yes	K-means – 2 clusters		Overlap of each cluster to produce 16 habitats		All three habitats associated with survival present in high enhancing segment One habitat predictive of survival occurred in high FLAIR/high T1+C intensity One habitat in very periphery, two in enhancing core	One habitat predictive of survival correlated with necrosis quantification	Habitats associated with various pathways including NFkB, DNA damage response/transduction and STAT1/NK activation
You 2018 Michigan, USA (27)	Glioblastoma N = 21	Post-resection pre-radiotherapy	T1 T1+C T2 FLAIR DWI ADC FA CBV kTrans MET PET	Yes	Hierarchical clustering to derive habitats				5/10 patients had recurrence related to MET only area (metabolically active rim)	1. High T2/FLAIR habitat mostly in necrotic core 2. High DWI habitat surrounded FLAIR region – core cellular component 3. High methionine habitat in periphery – metabolically active rim	
Stringfield 2019 Multi-centre (28)	Glioblastoma N = 74 (37 in each cohort of long and short term survivors)	Preoperative	T1 T1+C FLAIR	Yes	Otsu thresholding – 2 (FLAIR) or 3 (T1+C) clusters			Overlap to produce 6 habitats	High FLAIR/high T1c habitat present in significantly higher volume in long term survivors		
Li 2019 Cambridge, UK (29)	Glioblastoma Maximal resection Performance status 0-1 N = 112	Preoperative	ADC rCBV	Yes	Quartiles – 4 clusters each			Interested in two habitats – lowest quartile rCBV/lowest quartile ADC, lowest quartile rCBV/highest quartile ADC	Higher volume of these habitats associated with better PFS Lactate/Creatine ratio in these regions associated with shorter PFS and OS	Minimally invasive phenotype defined on DTI had lower proportion of the low rCBV/low ADC habitat	
Alvarez-Torres 2019 Multi-centre (30)	Glioblastoma (N = 184)	Preoperative	rCBV rCBF	NR	Gaussian mixture modelling to yield 4 habitats per patient				Several habitats predictive of survival		
Park & Kim 2020-2021 Seoul, South Korea (31, 32)	Glioblastoma (IDH wildtype; various N)	Post chemo-radiotherapy	T1+C ADC rCBV +/- EPT	NR	K-means clustering to define various habitats using combinations of ADC, rCBV and EPT				Hypovascular cellular habitat (low rCBV and ADC) and hypovascular low conductivity (low rCBV and EPT) habitats strongly correlated with site of future progression.		
Xu 2021 New-York USA (33)	Glioblastoma N = 263 from BraTS 2020 training dataset	Preoperative	T1+C T2 FLAIR	NR	Used simple linear interactive clustering (SLIC) – method that depends on intensity of pixels and their location				Graph features of habitats improved overall survival cox regression model		
Bailo 2022 Milan, Italy (34)	High grade gliomas (including 12 glioblastomas)	Preoperative	Vp MD FAZA PET	NR	Otsu thresholding to identify high/low regions of each biomarker in enhancing tumour or oedema	Multiple overlaps between clusters – 8 habitats possible				Habitats with high Vp/high FAZA uptake (regardless of MD) correlated with hyperplastic vessels and cellularity with low rate of necrosis. Largest volumetric representation was by ‘less aggressive’ habitats comprising low Vp/low FAZA uptake. These correlated with low cellularity and no signs of necrosis/angiogenesis.	
Yang 2022 Xi’an, China (9)	Glioblastoma (test cohort of 122, validation cohort of 65 patients)	Preoperative	T1 T1+C T2 FLAIR	Yes	Investigated oedema region only. Determined optimal number of K-means clusters using elbow plot method. Performed K-means clustering and produced 4 habitats.				Defined high risk habitat in oedema region based on radiomic features. This habitat improved performance of cox regression model of overall survival.		High risk habitat not correlated with MGMT methylation status

This table summarises data from 15 studies that have performed habitat imaging in glioblastoma. T1 + C, T1 with contrast; FLAIR, Fluid Attenuated Inversion Recovery; ADC, Apparent Diffusion Coefficient; DWI, Diffusion Weighted Imaging; FA, Fractional Anisotropy; CBV, cerebral blood volume; MET PET, methionine positron emission tomography; FAZA PET, 8F-labeled fluoroazomycinarabinoside PET, localises to hypoxic regions; EPT, Electrical Properties Tomography imaging.

The one step approach to deriving habitats is akin to clustering across a genomic dataset. You et al. derived habitats using hierarchical clustering in 21 glioblastoma patients and were able to relate these to survival and tumour biology descriptively. They found three main biomarker clusters that were named based on the most clinically relevant biomarker of the group: ‘FLAIR’ cluster – FLAIR, quantitative T1 and T2 signal, and ADC; MET cluster - methionine positron emission tomography (MET PET; methionine is an amino acid PET tracer that localises to metabolically active tumour), CBV and *K* ^Trans^ (marker of permeability; defined in **Table 1**); and diffusion-weighted imaging (DWI) cluster – DWI and fractional anisotropy (FA; defined in **Table 1**). The necrotic core was represented by the FLAIR cluster, surrounded by the core cellular component represented by both the FLAIR and DWI clusters, in turn encircled by a metabolically active rim represented by the methionine cluster. This method was therefore able to capture a degree of glioblastoma’s heterogeneity. The clinical utility of their method was limited in detecting tumour recurrence, localised to the methionine cluster in only 5 out of 10 cases (27).

Other studies have also found utility in applying the one-step approach. Juan-Albarracín et al. developed an automated method of habitat generation using gaussian mixture modelling applied to rCBV and relative cerebral blood flow (rCBF; defined in **Table 1**) to produce four habitats – two in the enhancing core (high/low angiogenic) and two in the oedema (infiltrated and vasogenic). They demonstrated that the median rCBVmax or rCBFmax values in the high and low angiogenic habitats were predictive of survival (35). These findings were subsequently validated in a multi-centre study (30). This group has made their technique to generate habitats into a standardised and adaptable pipeline for other centres (36).

The two step approach to generating habitats is more commonly used in the literature (16, 22–26, 28, 29, 34). The first step of biomarker clustering itself can be done using simple methods, such as by dividing intensity values based on average values/quartiles, or using machine learning methods. Habitats are then visualised as the overlaps of individual biomarker clusters. For example, Lee et al. used this approach in 74 glioblastoma patients from The Cancer Genome Atlas (TCGA), using Gaussian mixture modelling to cluster biomarkers (enhancement on T1 with contrast and FLAIR), which were then used to produce four habitats. Spatial features of these habitats were predictive of survival and had a high sensitivity for predicting glioblastoma transcriptomic subtype – highest for the proneural subtype with an area under curve value of 93% (22).

Only few studies have attempted to relate habitats to specific histological or molecular signatures (**Table 2**) (25, 26, 28, 29). Dextraze et al. analysed 85 glioblastoma patients from TCGA, and reported that the volume of a habitat localised to necrotic regions was positively correlated with an upregulation in Nuclear factor kappa B signaling, for example (26). Bailo et al. is the only study that attempted to directly sample characterised habitats (34). They studied 17 high grade glioma patients and used the two step approach with three biomarkers: *v_p_* - plasma volume (a DCE-MRI biomarker; defined in **Table 1**), mean diffusivity (defined in **Table 1**) and uptake of a PET tracer that localizes to hypoxic regions. They undertook multi-regional tumour sampling and related habitats to histopathological features. Although conclusions were difficult to draw in view of the sample size, they identified habitats correlated with more aggressive histological features such as high cellularity and neovascularization (34).

In summary, current experience with habitat imaging in glioblastoma has mostly focused on the prognostic value of the technique applied to preoperative imaging and, in general, correlating habitat frequencies with global tumour biological features/molecular pathways. Existing studies have not explored technical considerations that are of critical importance to habitat imaging and its validation, including derivation method, biomarker selection, imaging acquisition parameters and tissue sampling. These will be explored in the following sections to provide a framework for future studies.

### Technical considerations

There are several technical considerations of relevance to habitat imaging.

***One versus two step approach***. Habitat generation has been described using two main methods (**Figure 2**). The one step approach clusters data from multiple imaging biomarkers directly, whereas the two step approach has an intermediate clustering step for each imaging biomarker selected. Both techniques are dependent on accurate image registration, which refers to the process of aligning different MRI scans. Quantitative maps represent imaging biomarker values on each pixel (instead of signal intensity) and are produced from MRI sequences to which they are inherently aligned (e.g. an ADC/rCBV image is aligned to the DWI/dynamic susceptibility contrast MRI from which it was derived, respectively). When biomarker values are extracted from a three-dimensional image to a two-dimensional table of data for clustering, they are done so in pixel-order (i.e. from one edge of the image to the other), which will differ from sequence to sequence due to differences in resolution and therefore the number of potential ‘rows of data’. In order to correct for this, image registration is performed to spatially align and transform two images, but this results in distortion of individual values. The newly aligned images will include pixel values that were not present in the original data-set but derived from neighbouring values through interpolation (37). It is not ideal to register the whole quantitative map therefore as it creates artificial data values and can amplify artifacts, but this has been universally done in studies utilising the one step approach to habitat imaging (34). A workaround is to use multiple imaging biomarkers from the same MRI sequence – such as with dynamic contrast enhanced (DCE) MRI (demonstrated in **Figure 2** – top panel). DCE-MRI offers a multitude of imaging biomarkers that can assess several aspects of brain tumour physiology including vessel permeability (*K* ^Trans^), vascularity (*v_p_* - plasma volume), blood flow and cell density (*v_e_* - extravascular extracellular space) (38). These have been validated for use in other brain tumours such as vestibular schwannomas (38), though DCE-MRI is under-utilised in glioblastoma literature (39). Another solution is to use the two step approach, which clusters each biomarker individually prior to habitat generation. Registration is done on clustered data which minimises the effect of extreme/artifactual values (which would otherwise be present in the up-scaled, registered data at a higher frequency). It is also predominantly the edge voxels between different clusters that are affected by spatial transformation steps. A comparison between the one and two step techniques is required in future studies.

***Biomarker choice***. A significant limitation of most existing studies is their reliance on imaging biomarkers derived from non-quantitative, structural MRI sequences (e.g. T1- and T2-weighted MRI). These sequences were developed for visualisation of gross anatomy and for this purpose, there is a high degree of consistency in brain structural morphology (40). However, their signal intensity values are affected by hardware factors, such as magnetic field strength inhomogeneity, head placement within the receiver coil, image intensity scaling factors and image acquisition parameters (41). It is difficult to completely negate these effects or correct them using a normalisation step (41). Habitats derived from functional MRI sequences (e.g. diffusion/perfusion) have demonstrated the greatest external validity and this approach should therefore be favoured (30). The functional imaging biomarkers to consider for habitat generation depend on the purpose of the exercise. If this is a clinical aim, such as the identification of treatment-resistant habitats, then robust biomarkers of cellularity and perfusion are important. The imaging biomarkers should also be readily available across centres to allow external validation/adoption. In this case, we hypothesise that ADC, rCBV and *K* ^Trans^ are good candidates to further explore, given their sensitivity to treatment-related change and widespread use (42).

***Biomarker calculation***. The calculation method is an important consideration for functional imaging biomarkers. For example, the numerical value of DCE-MRI biomarkers such as *K* ^Trans^ can vary in the same dataset depending on the pharmacokinetic model used, due to different underlying physiological assumptions (43). For DCE-MRI analysis in glioblastoma, an extended Toft’s model is usually employed that models contrast leakage between intra/extravascular tissue compartments, modified (‘extended’) for appropriate contribution of the intravascular compartment (44). This can be combined with new processing techniques such as the Legatos method, described and validated by our group, which combines high temporal and high spatial resolution DCE-MRI data, to facilitate habitat imaging (used for **Figure 2** top panel) (38, 45). Different model assumptions also also apply to diffusion-derived biomarkers such as ADC, which can be defined using a mono-exponential model (fits a straight line through a graph of signal intensity versus b-values - usually 0 and 1000 s/mm^2^; b-values denote the strength of the magnetic field gradient applied in diffusion MRI studies) and more complex exponential models (fits a more complex function involving multiple b-values), with the latter typically producing more accurate results (46). However, to date, studies using ADC for habitat imaging have used monoexponential models (16, 27). The Quantitative Imaging Biomarkers Alliance (QIBA) is an ongoing effort that aims to produce standards for use of specialist imaging such as diffusion/perfusion MRI in clinical and research environments (47). This work could help to standardise biomarker calculation methods, which are currently diverse, poorly understood and not robustly validated (47). As an example, although rCBV is frequently cited in glioblastoma literature, the variation in how it is derived is often not acknowledged. Indeed, it can be derived from dynamic susceptibility contrast enhanced MRI (DSC-MRI) using almost any major imaging analysis software package (including FMRIB Software Library, 3D slicer^©^, Matlab^©^ and Osirix^©^) and each uses a different calculation method (48). In general, we recommend utilisation of robust biomarker calculation methods that are amenable to external uptake and therefore validation.

***Image acquisition*.** Habitat generation requires a relatively high spatial resolution. This should be small enough with respect to the size of the tumour to avoid partial volume effects, which occur with larger voxel sizes (i.e. thicker MRI slices) that average MR signal from multiple tissue components included in each voxel (49). However, there is a trade-off between spatial resolution and the signal-to-noise ratio (SNR) that is needed for accurate estimation of imaging biomarker values on a voxel-level, like ADC and *K* ^trans^ (47, 50). The SNR is proportional to voxel volume as larger voxels contain a higher number of protons that subsequently produce a greater MR signal (49). The imaging time must also be considered, as longer durations can result in motion artifacts. Initiatives like QIBA provide guidance for the acquisition of quantitative imaging biomarkers and the use of such MR acquisition parameters would allow a more robust comparison of habitats across centres, especially in the case of multi-centre tissue sampling (47). Scan angulation is another important consideration for the two step approach to remain consistent between functional and anatomical sequences (50). Where data from multiple acquisitions is being utilised, if scan angulations are not aligned, then potentially all data will be resampled and interpolated during image registration. In summary, habitat imaging requires a relatively high spatial resolution (2-3.5mm slice thickness in our experience) that preserves the SNR, and utilises sequences with relatively consistent angulation to structural sequences.

**Figure 3:**
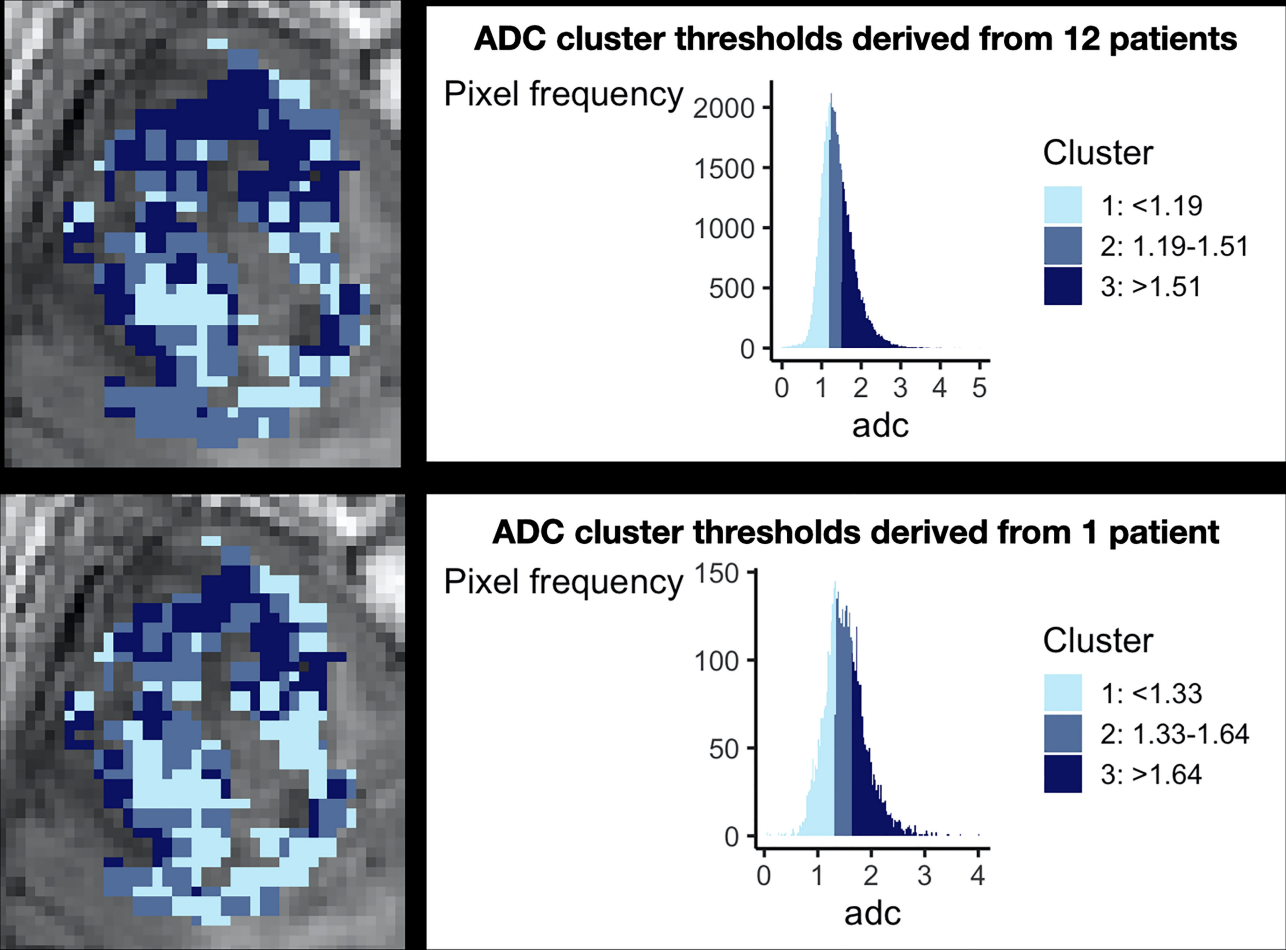
The importance of considering group level data during clustering. This figure demonstrates the necessity of combining patient data for clustering. The top panel shows preoperative ADC data from 12 glioblastoma patients after clustering, demonstrating a histogram with a smooth gaussian shape. The bottom panel shows the results of clustering when data from only one individual patient is considered, revealing a more irregular histogram and different cut off values for each cluster. Corresponding cluster regions are displayed visually on the left of each panel. This technical consideration is of particular importance as it has implications for prospective habitat generation in validation cohorts, which is dependent on robust predefined cut offs.

***Individual versus group level data***. This is of particular relevance to glioblastoma given its significant inter-patient heterogeneity. The techniques described above consider imaging biomarker data on an individual patient level. Biomarker clustering is performed using threshold values defined per patient, rather than the larger group. This is largely because they utilize structural MRI sequences alone, which are not validated for scaled comparisons between patients. However, for quantitative imaging biomarkers, this is an important consideration for glioblastoma given its significant inter-patient heterogeneity. In our previous meta-analysis for example, the mean tumoral blood flow relative to normal appearing white matter across glioblastoma patients in the literature varied from 1.6-7.9 (39). To demonstrate the importance of group-level data, **Figure 3** demonstrates differences in ADC thresholds when clustering is performed at the individual patient versus group level. The advantage of group-level definitions is that they allow for reproducibility across both retrospective and prospective datasets. Group-level definitions should therefore be utilised in future studies.

***Machine learning***. Unsupervised machine learning techniques can be used for clustering purposes. Studies have used two main approaches:

- Imaging sciences approaches: Otsu thresholding analyses the distribution of pixel intensity values to determine threshold value(s) to maximise discrimination between (usually two) pixel classes (51). This has been utilised in studies using structural as well as quantitative MRI sequences (25, 28, 34). Both commercial and open-source software packages are also capable of applying predetermined thresholds to automatically segment a region of interest into different classes. For example, the open-source FMRIB’s Automated Segmentation Tool (‘FAST’) can segment brain images into white matter, grey matter and cerebrospinal fluid (52). Although this tool was used in one study investigating glioblastoma habitats, it is not designed to segment tumour regions (24). These techniques are usually applied to individual MRI sequences and do not therefore account for inter-patient heterogeneity.
- Classical approaches: k-means clustering separates data into clusters by iteratively allocating data points to cluster ‘centroids’ (numerical points that represent a group of adjacent data points) and updating centroids to minimise the sum of squared distances between data points and corresponding cluster centroids to which they are assigned. This algorithm is very simple and efficient, but sensitive to extreme values, given its reliance on the mean for centroids. It also requires the user to specify the number of clusters required (53). Most existing studies using k-means clustering to generate habitats have not described how the number of clusters (i.e. habitats) was determined (26). This typically requires additional analysis such as the within cluster sum-of-squared or ‘Elbow plot’ method (54). This method plots the number of clusters (x-axis) against the sum of squared distance between each point and the centroid (y-axis). The optimal number of clusters is the point of maximal ‘bend’ or ‘elbow’ (33, 54). Gaussian mixture modelling clusters data by identifying gaussians (i.e. normal distributions) within the data distribution and it can perform hard or soft clustering of data to different gaussians (53). The presence of multiple gaussians is therefore an assumption of this technique, which may not be accurate. For example, **Figure 3** shows only one smooth ADC gaussian when data from all patients is considered. Hierarchical clustering groups together datapoints based on local proximity (53). This method has only been applied to individual patient data rather than group-level data, for which it was designed (27).

In summary therefore, the use of machine learning techniques to generate imaging habitats in glioblastoma requires further evaluation using robust methodology. In particular, studies should justify the number of clusters selected, rather than basing this figure on an arbitrary value. The role of machine learning techniques should also be clarified through comparison to simpler techniques such as ‘binning’ of data-values into clusters based on quartile or mean values.

***Deep-learning***. Deep learning (DL) is a subfield of machine learning that is capable of learning which features are most relevant for classification/clustering problems. It is classically described in three stages (55, 56):

1. Input of labelled training data – this is high-dimensional data, which has been assigned labels manually. For example, for a tumour segmentation task, this may be pixels assigned as tumour or brain.
2. Development of neural network – this comprises an input layer, one or more hidden layers and an output layer. In simple terms, the input is mathematically mapped to the output by a series of functions (contained in hidden layers) that try to model the relationship between the two. In imaging research, a ‘convolutional’ neural network (CNN) is typically used which applies an additional convolution function (also referred to as a kernel) to the input to provide an estimation of spatial relationships (55).
3. Validation of neural network – this step utilises an additional validation dataset to validate the neural network that has been developed.

DL can be implemented in habitat imaging pipelines in at least three ways:

1. Tumour segmentation: habitat imaging requires accurate three-dimensional delineation of the tumour and/or peritumoral oedema, to allow precise monitoring of longitudinal changes and treatment planning (57). Manual segmentation is time consuming and subjective, even in expert hands, with a high inter-rater variability. This was best illustrated in the Multimodal Brain Tumour Image Segmentation Benchmark (BRATS) challenge, which compared glioma segmentation algorithms against expert labelling. The authors found a high degree of disagreement between human raters (58). Approaches utilising a CNN can achieve/exceed performance of experts. For example, in habitat imaging, the ONCOhabitats algorithm proposed by Juan- Albarracín et al. utilises an initial segmentation step incorporating a CNN. The authors developed this utilising 210 high grade glioma scans from the BRATS dataset, basing segmentation on structural sequences (T1 pre and post gadolinium, T2-weighted and FLAIR-MRI). Their method achieved a high sensitivity of up to 87% for whole tumour, and very high specificity of 99% for all tumour regions (enhancing tumour/oedema/whole tumour) (36).
2. Pharmacokinetic model fitting: this is of relevance to techniques such as DCE-MRI. Traditionally, a non-linear least square (NLLS) method is used to fit pharmacokinetic models to the four-dimensional data obtained from DCE-MRI (i.e. 3D volumes acquired serially with time). DL methods such as CNN can produce more precise parameter estimates with less noise, although they are also prone to systematic errors (59).
3. Habitat generation: a difficulty in using DL for habitat generation in glioblastoma is its reliance on labelled data and as a result, DL has not yet been used for this purpose. A comprehensive reference resource with labelled habitats would facilitate the development of robust DL methods for habitat generation (see Discussion).

In summary, DL is an evolving and exciting field, whose methodology could be incorporated into the first arm of habitats pipeline to allow semi-automated tumour segmentation. However, at present, experience with DL is limited and its role remains to be defined.

***Habitat volume and sampling***. This factor is of particular clinical relevance to validate habitat methodology. In theory, any number of imaging biomarkers can be clustered and combined to produce habitats. However, an increasing number of biomarkers and biomarker clusters decreases the habitat volume limiting the possibility to cross validate the habitat with tumour sampling. Furthermore, the conceptual meaning of habitats may decrease the more biomarkers are used to define them. Bailo et al. utilised image-guided biopsies to sample habitats derived from three biomarkers clustered into two categories each (low/high). However, only 19/31 biopsies they performed contained a single habitat, whereas others contained multiple habitats (34). This would suggest that even fewer biomarkers should be utilised to allow a large enough volume to allow accurate histological sampling. In the context of glioblastoma, this should be at least 1 mm^3^, which is the minimum volume of a brain biopsy (60). In reality, the sampled area is likely to be even larger than this and therefore, without adjusting habitat size, sampling of tissue will include multiple habitats that will confound results. Relating habitats to autopsy specimens should be avoided as these may be obtained several months after the imaging study and sampled areas can be much larger than habitat (24). As habitat samples are likely to be small, efforts must be taken to preserve tumour cell viability. This includes transporting them on dry ice and fixing/snap freezing samples at the earliest possible convenience (61). An alternative strategy to validating habitats, as utilised in other cancers, relates to correlation with metabolic imaging such as PET, although this less widely available (62). In summary, for heterogenous tumours like glioblastoma, habitat sampling and validation is important to guide the development of personalised therapy.

These technical considerations highlight the need for future studies to evaluate divergent methodologies that are not fully explored, to provide reproducible habitats across centres.

### Clinical application

A robust and reproducible method of defining glioblastoma habitats has several clinical applications.

***Tumour sampling***. Habitat guided tumour sampling is possible as habitat maps can be imported into conventional neuronavigation software used in neurosurgical planning (34). This software is capable of image registration but is optimised for structural and functional MRI sequences. To avoid registration errors in this specialist setting, it is therefore important for the final habitat output map to be registered to a structural MRI sequence (typically T1 post gladolinium) prior to its export into neuronavigation software. It can then be used to direct surgical sampling. The location of intra-operative biopsies can be mapped back to MRI scans using the FMRIB Software Library’s upcoming Tensor Imaging Registration Library (TIRL) tool, which can act as a bridge between imaging and histopathology (79). Habitat-guided tumour sampling has the potential to reduce spatial heterogeneity between acquired specimens. Furthermore, in glioblastoma patients undergoing biopsy alone, which comprise around 40% of all cases (63), the tumoral yield could be increased by targeting more cellular habitats - with lower ADC values for example. This is also potentially advantageous for genomic sequencing analyses. Treatment resistant habitats could also be sampled, especially in the case of multi-focal and ‘butterfly’ glioblastomas (that cross the corpus callosum) where a surgical target for biopsy is not always clear.

***Diagnostics***. Habitat imaging provides an additional tool for radiologists to define a lesion’s imaging signature, which could aid diagnostics. This is of particular relevance at present given the increasing emphasis on early time point interventions for newly-diagnosed glioblastoma, including preoperative therapies, which may require imaging diagnosis alone (2). As an example, PreOperative Brain Irradiation in Glioblastoma (POBIG - NCT03582514) is an ongoing phase I trial (led by the senior author of this review) that will evaluate the safety and feasibility of preoperative radiotherapy in newly-diagnosed glioblastoma patients based on imaging diagnosis alone (64). Confirmation of diagnosis is of critical importance in preoperative treatment studies and some have implemented a first step of a pre-resection tumour biopsy to offset the risk of a misdiagnosis (65).

***Targeted treatment***. The habitat profile of a tumour may correlate with key molecular changes such as O^6^-methylguanine-DNA methyltransferase (MGMT) promotor methylation, which could non-invasively aid the selection of patients for future neoadjuvant trials (2, 66). In addition, interventional approaches would benefit from prior knowledge of habitats that have associated aggressive histopathological tumour signatures such hypoxia. Notably, this cancer hallmark is present both microscopically in tumour niches around palisading necrotic regions, but also macroscopically, in hypoperfused areas such as the peri-necrotic rim (8, 67). Treatment-resistant habitats could be targeted with regional dose-boost radiotherapy and/or surgical resection, such as in the case of butterfly lesions where there is discrepancy in surgical decision making (68). This is an important area to explore given the negative results from dose escalation based on structural imaging and the ongoing attempts to improve the outcome by escalating the dose in tumour areas identified on functional imaging (69–71). Habitat-guided radiotherapy dose boost is already being prospectively evaluated in prostate cancer (72). Dynamic assessment of habitat treatment response offers a more personalised approach that allows intensification of treatment only when required, on a regional basis (**Figure 1**).

## Discussion

Habitat imaging in glioblastoma has several potential clinical benefits and applications but there remain a number of technical challenges. Based on the imaging biomarker roadmap, suitable data does not currently exist to evaluate this strategy towards validation and more robust data is required (11).

Existing studies that have derived imaging habitats in glioblastoma patients and studied their associated histological/molecular characteristics are not comprehensive or sufficiently robust (26, 34). There are multiple technical considerations of relevance to both the process of imaging habitat generation and subsequent histological validation, that require further study. The methodology employed to generate imaging habitats should offer low variation/high repeatability within the same patient in the absence of clinical change when imaging is performed longitudinally. Such repeatability depends on the underlying imaging biomarkers selected and has been demonstrated for quantitative imaging biomarkers derived from diffusion and perfusion MRI (73, 74).

Habitat volume is a key challenge that should be overcome prior to histological validation. Multiple habitat inclusion in image-guided biopsies can lead to non-specific results. For example, the usual inverse correlation between mean diffusivity and cellularity was not observed in the study by Bailo et al., in which over one third of biopsies contained multiple habitats (34). Better characterisation of the biology of habitats could also pave the way for DL techniques to optimize habitat generation. As reviewed above, DL techniques depend on labelled data points. A comprehensive investigation that spatially links histopathological features (e.g. cellularity, perfusion and necrosis) to multi-modal imaging would allow a CNN to be trained that can provide parameter maps relating to these features. This step is of primary importance towards translation and clinical use of habitat imaging, which is otherwise time consuming and reliant on specialist software/expertise.

Habitat imaging in other cancer types including breast, prostate and sarcoma has reached histological or preclinical validation, and even clinical use (62, 72, 75). Some experiences have utilised additional strategies to those reviewed above that merit discussion. Xing et al. described an initial step of qualitatively defining five habitats based on radiologists’ assessment of T2/diffusion weighted MRI in 18 patients with biopsy-proven soft tissue sarcoma. As a second step, they then utilised gaussian mixture modelling to create quantitative definitions that described the probability of a pixel belonging to one of these specific habitats. This approach identified a validated necrotic habitat that correlated well with preoperative fluorodeoxyglucose-(FDG)-PET, which increased after preoperative radiotherapy (62). Another approach utilised in a preclinical sarcoma mouse model involves registering fine cut tissue sections to multiparametric imaging, to identify imaging signatures predictive of specific histologically defined habitats (76). This is challenging in glioblastoma patients as it requires *en-bloc* resection, which is only feasible in limited locations within the brain and in most cases will not capture infiltrative components of the tumour periphery (77), or the availability of temporally correlated post-mortem specimens. However, unlike experiences in glioblastoma patients, both of these strategies limit the number of imaging habitats to those apparent clinically.

Understanding the biological meaning of habitats is of direct clinical importance and experience in prostate cancer has demonstrated its value. Stoyanova et al. defined habitats based on DCE/ADC MRI and correlated them with Gleason scores on finely cut prostate cancer sections. Their prior work had identified thresholds based on DCE/ADC that correlated with higher Gleason scores. They identified a habitat that correlated well with a Gleason score of ≥7, representing increased likelihood of cancerous tissue, with an area under curve of 0.8. This habitat is now being prospectively targeted with regional dose boost radiotherapy in a phase II randomised trial (72).

A limitation of concepts presented in this work is reliance on relatively small studies with largely un-validated methodologies. There is wide scope for refinement and validation of imaging habitat techniques in glioblastoma patients specifically given that firstly, multiparametric MRI is a standard of care, and secondly that MRI-guided surgery is routine in the brain (78). Future studies should therefore focus on histologically validating robustly-derived imaging habitats. A generic limitation of studies in other cancer types is the lack of real-time tissue sampling from habitats and reliance on registration of histological sections with imaging. This is not always reliable, given the gantry angle of MRI machines and potential for tissue distortion during slice extraction and preparation.

## Conclusion

Habitat imaging is a relatively novel concept that reflects the evolution of imaging biomarkers, to potentially offer a superior means to assess tumour biology and response to treatment in glioblastoma. At present, literature is limited and further studies are required to both robustly generate and validate this technique. This is an important area of research given the multiple clinical applications of habitat imaging that could facilitate more personalised therapy Glioblastoma. Future studies should investigate clustering techniques (machine learning vs. simpler strategies), choice of imaging biomarkers, habitat reproducibility/external validity and means to histologically validate findings, towards the common goal of identifying strategies to overcome treatment-resistance of habitat defined regions.

## Data availability statement

All datasets presented in this study are included in the article/**Supplementary Material**.

## Author contributions

MW: drafted manuscript, performed literature review, aided with figures. PVH: drafted manuscript, aided in literature review, reviewed manuscript. EH: aided in literature review and reviewing manuscript draft. K-LL and XZ: produced tables and data for figures, reviewed manuscript draft. AJ: designed concept, reviewed manuscript. MI: oversaw machine learning sections, reviewed manuscript draft. JO’C: designed concept, aided in processing figures, reviewed manuscript. ID: designed concept, reviewed manuscript. UVH: designed concept, reviewed manuscript. DC and GB: formulated concept of study, obtained original data for figures, oversaw manuscript editing and finalization. All authors contributed to the article and approved the submitted version.

## Acknowledgments

The authors acknowledge Istvan Huszar (contributor to the FMRIB Software Library) from the University of Oxford for his assistance with image registration.

## Conflict of interest

The authors declare that the research was conducted in the absence of any commercial or financial relationships that could be construed as a potential conflict of interest.

## Publisher’s note

All claims expressed in this article are solely those of the authors and do not necessarily represent those of their affiliated organizations, or those of the publisher, the editors and the reviewers. Any product that may be evaluated in this article, or claim that may be made by its manufacturer, is not guaranteed or endorsed by the publisher.

## Supplementary material

Supplementary Figure 1 Search strategy. To review the current evidence on imaging habitats, MEDLINE was queried systematically using the search terms described by two independent authors (MW, PVH) with discrepancies resolved through discussion with a third (EH). A total of 15 articles were included in the final review relating to imaging habitats that are summarised in .

## References

[1] Stupp R Mason WP Van Den Bent MJ Weller M Fisher B Taphoorn MJ . Radiotherapy plus concomitant and adjuvant temozolomide for glioblastoma. N Engl J Med (2005) 352(10):987–96. doi: 10.1056/NEJMoa043330 15758009

[2] Waqar M Roncaroli F Lehrer EJ Palmer JD Villanueva-Meyer J Braunstein S . Early therapeutic interventions for newly diagnosed glioblastoma: Rationale and review of the literature. Curr Oncol Rep (2022) 24(3):311–24. doi: 10.1007/s11912-021-01157-0 PMC8885508 35119629

[3] Waqar M Trifiletti DM Mcbain C O'connor J Coope DJ Akkari L . Rapid early progression (REP) of glioblastoma is an independent negative prognostic factor: Results from a systematic review and meta-analysis. Neurooncol Adv (2022) 4(1):vdac075. 35769410 10.1093/noajnl/vdac075 PMC9234755

[4] Barthel FP Johnson KC Varn FS Moskalik AD Tanner G Kocakavuk E . Longitudinal molecular trajectories of diffuse glioma in adults. Nature (2019) 576(7785):112–20. doi: 10.1038/s41586-019-1775-1 PMC6897368 31748746

[5] Sottoriva A Spiteri I Piccirillo SG Touloumis A Collins VP Marioni JC . Intratumor heterogeneity in human glioblastoma reflects cancer evolutionary dynamics. Proc Natl Acad Sci U S A (2013) 110(10):4009–14. doi: 10.1073/pnas.1219747110 PMC3593922 23412337

[6] Ravi VM Will P Kueckelhaus J Sun N Joseph K Salie H . Spatially resolved multi-omics deciphers bidirectional tumor-host interdependence in glioblastoma. Cancer Cell (2022) 40(6):639–55.e13. doi: 10.1016/j.ccell.2022.05.009 35700707

[7] Aderetti DA Hira VVV Molenaar RJ and Van Noorden CJF . The hypoxic peri-arteriolar glioma stem cell niche, an integrated concept of five types of niches in human glioblastoma. Biochim Biophys Acta Rev Cancer (2018) 1869(2):346–54. doi: 10.1016/j.bbcan.2018.04.008 29684521

[8] Lam KHB Leon AJ Hui W Lee SC Batruch I Faust K . Topographic mapping of the glioblastoma proteome reveals a triple-axis model of intra-tumoral heterogeneity. Nat Commun (2022) 13(1):116. doi: 10.1038/s41467-021-27667-w 35013227 PMC8748638

[9] Yang Y Han Y Zhao S Xiao G Guo L Zhang X . Spatial heterogeneity of edema region uncovers survival-relevant habitat of glioblastoma. Eur J Radiol (2022) 154:110423. doi: 10.1016/j.ejrad.2022.110423 35777079

[10] Choi SW Cho HH Koo H Cho KR Nenning KH Langs G . Multi-habitat radiomics unravels distinct phenotypic subtypes of glioblastoma with clinical and genomic significance. Cancers (Basel) (2020) 12(7). doi: 10.3390/cancers12071707 PMC7408408 32605068

[11] O'Connor JP Aboagye EO Adams JE Aerts HJ Barrington SF Beer AJ . Imaging biomarker roadmap for cancer studies. Nat Rev Clin Oncol (2017) 14(3):169–86. doi: 10.1038/nrclinonc.2016.162 PMC5378302 27725679

[12] Li KL Djoukhadar I Zhu X Zhao S Lloyd S Mccabe M . Vascular biomarkers derived from dynamic contrast-enhanced MRI predict response of vestibular schwannoma to antiangiogenic therapy in type 2 neurofibromatosis. Neuro-oncology (2016) 18(2):275–82. doi: 10.1093/neuonc/nov168 PMC4724182 26311690

[13] Singh G Manjila S Sakla N True A Wardeh AH Beig N . Radiomics and radiogenomics in gliomas: a contemporary update. Br J Cancer (2021) 125(5):641–57. doi: 10.1038/s41416-021-01387-w PMC8405677 33958734

[14] Gillies RJ Kinahan PE Hricak H . Radiomics: Images are more than pictures, they are data. Radiology (2016) 278(2):563–77. doi: 10.1148/radiol.2015151169 PMC4734157 26579733

[15] Xie C Yang P Zhang X Xu L Wang X Li X . Sub-Region based radiomics analysis for survival prediction in oesophageal tumours treated by definitive concurrent chemoradiotherapy. EBioMedicine (2019) 44:289–97. doi: 10.1016/j.ebiom.2019.05.023 PMC6606893 31129097

[16] Khalifa J Tensaouti F Lotterie JA Catalaa I Chaltiel L Benouaich-Amiel A . Do perfusion and diffusion MRI predict glioblastoma relapse sites following chemoradiation? J Neurooncol (2016) 130(1):181–92. doi: 10.1007/s11060-016-2232-8 27502603

[17] Napel S Mu W Jardim-Perassi BV Aerts H Gillies RJ . Quantitative imaging of cancer in the postgenomic era: Radio(geno)mics, deep learning, and habitats. Cancer (2018) 124(24):4633–49. doi: 10.1002/cncr.31630 PMC6482447 30383900

[18] Spence AM Muzi M Swanson KR O'sullivan F Rockhill JK Rajendran JG . Regional hypoxia in glioblastoma multiforme quantified with [18F]fluoromisonidazole positron emission tomography before radiotherapy: Correlation with time to progression and survival. Clin Cancer Res (2008) 14(9):2623–30. doi: 10.1158/1078-0432.CCR-07-4995 PMC4415875 18451225

[19] Pruis IJ Koene SR Van Der Voort SR Incekara F Vincent A Van Den Bent MJ . Noninvasive differentiation of molecular subtypes of adult nonenhancing glioma using MRI perfusion and diffusion parameters. Neurooncol Adv (2022) 4(1):vdac023. doi: 10.1093/noajnl/vdac023 35300151 PMC8923005

[20] Lee DH Park JE Kim N Park SY Kim YH Cho YH . Tumor habitat analysis by magnetic resonance imaging distinguishes tumor progression from radiation necrosis in brain metastases after stereotactic radiosurgery. Eur Radiol (2022) 32(1):497–507. doi: 10.1007/s00330-021-08204-1 34357451

[21] O'Connor JP Rose CJ Waterton JC Carano RA Parker GJ Jackson A . Imaging intratumor heterogeneity: role in therapy response, resistance, and clinical outcome. Clin Cancer Res (2015) 21(2):249–57. doi: 10.1158/1078-0432.CCR-14-0990 PMC4688961 25421725

[22] Lee J Narang S Martinez J Rao G Rao A . Spatial habitat features derived from multiparametric magnetic resonance imaging data are associated with molecular subtype and 12-month survival status in glioblastoma multiforme. PloS One (2015) 10(9):e0136557. doi: 10.1371/journal.pone.0136557 26368923 PMC4569439

[23] Lee J Narang S Martinez JJ Rao G Rao A . Associating spatial diversity features of radiologically defined tumor habitats with epidermal growth factor receptor driver status and 12-month survival in glioblastoma: Methods and preliminary investigation. J Med Imaging (Bellingham) (2015) 2(4):041006. doi: 10.1117/1.JMI.2.4.041006 26835490 PMC4718420

[24] McGarry SD Hurrell SL Kaczmarowski AL Cochran EJ Connelly J Rand SD . Magnetic resonance imaging-based radiomic profiles predict patient prognosis in newly diagnosed glioblastoma before therapy. Tomography (2016) 2(3):223–8. doi: 10.18383/j.tom.2016.00250 PMC5074084 27774518

[25] Zhou M Chaudhury B Hall LO Goldgof DB Gillies RJ Gatenby RA . Identifying spatial imaging biomarkers of glioblastoma multiforme for survival group prediction. J Magn Reson Imaging (2017) 46(1):115–23. doi: 10.1002/jmri.25497 27678245

[26] Dextraze K Saha A Kim D Narang S Lehrer M Rao A . Spatial habitats from multiparametric MR imaging are associated with signaling pathway activities and survival in glioblastoma. Oncotarget (2017) 8(68):112992–3001. doi: 10.18632/oncotarget.22947 PMC5762568 29348883

[27] You D Kim MM Aryal MP Parmar H Piert M Lawrence TS . Tumor image signatures and habitats: A processing pipeline of multimodality metabolic and physiological images. J Med Imaging (Bellingham) (2018) 5(1):011009. 29181433 10.1117/1.JMI.5.1.011009 PMC5686431

[28] Stringfield O Arrington JA Johnston SK Rognin NG Peeri NC Balagurunathan Y . Multiparameter MRI predictors of long-term survival in glioblastoma multiforme. Tomography (2019) 5(1):135–44. doi: 10.18383/j.tom.2018.00052 PMC6403044 30854451

[29] Li C Yan JL Torheim T Mclean MA Boonzaier NR Zou . Low perfusion compartments in glioblastoma quantified by advanced magnetic resonance imaging and correlated with patient survival. Radiother Oncol (2019) 134:17–24. doi: 10.1016/j.radonc.2019.01.008 31005212 PMC6486398

[30] Del Mar Alvarez-Torres M Juan-Albarracin J Fuster-Garcia E Bellvis-Bataller F Lorente D Reynes G . Robust association between vascular habitats and patient prognosis in glioblastoma: An international multicenter study. J Magn Reson Imaging (2020) 51(5):1478–86. doi: 10.1002/jmri.26958 31654541

[31] Park JE Kim HS Kim N Kim YH Kim JH Kim E . Low conductivity on electrical properties tomography demonstrates unique tumor habitats indicating progression in glioblastoma. Eur Radiol (2021) 31(9):6655–65. doi: 10.1007/s00330-021-07976-w 33880619

[32] Park JE Kim HS Kim N Park SY Kim YH Kim JH . Spatiotemporal heterogeneity in multiparametric physiologic MRI is associated with patient outcomes in IDH-wildtype glioblastoma. Clin Cancer Res (2021) 27(1):237–45. doi: 10.1158/1078-0432.CCR-20-2156 33028594

[33] Xu X Samaras D Prasanna P . Radiologically defined tumor-habitat adjacency as a prognostic biomarker in glioblastoma. Annu Int Conf IEEE Eng Med Biol Soc (2021) 2021:3998–4001. doi: 10.1109/EMBC46164.2021.9629779 34892107

[34] Bailo M Pecco N Callea M Scifo P Gagliardi F Presotto L . Decoding the heterogeneity of malignant gliomas by PET and MRI for spatial habitat analysis of hypoxia, perfusion, and diffusion imaging: A preliminary study. Front Neurosci (2022) 16:885291. doi: 10.3389/fnins.2022.885291 35911979 PMC9326318

[35] Juan-Albarracin J Fuster-Garcia E Perez-Girbes A Aparici-Robles F Alberich-Bayarri A Revert-Ventura A . Glioblastoma: Vascular habitats detected at preoperative dynamic susceptibility-weighted contrast-enhanced perfusion MR imaging predict survival. Radiology (2018) 287(3):944–54. doi: 10.1148/radiol.2017170845 29357274

[36] Juan-Albarracin J Fuster-Garcia E Garcia-Ferrando GA Garcia-Gomez JM . ONCOhabitats: A system for glioblastoma heterogeneity assessment through MRI. Int J Med Inform (2019) 128:53–61. doi: 10.1016/j.ijmedinf.2019.05.002 31160012

[37] Jenkinson M Chappell M . Introduction to neuroimaging analysis. 1st ed. New York, NY: Oxford University Press (2018) xvii(258).

[38] Lewis D Roncaroli F Agushi E Mosses D Williams R Li KL . Inflammation and vascular permeability correlate with growth in sporadic vestibular schwannoma. Neuro-oncology (2019) 21(3):314–25. doi: 10.1093/neuonc/noy177 PMC6380424 30388263

[39] Waqar M Lewis D Agushi E Gittins M Jackson A Coope D . Cerebral and tumoral blood flow in adult gliomas: A systematic review of results from magnetic resonance imaging. Br J Radiol (2021) 94(1125):20201450. doi: 10.1259/bjr.20201450 34106749 PMC9327770

[40] McGuire SA Wijtenburg SA Sherman PM Rowland LM Ryan M Sladky JH . Reproducibility of quantitative structural and physiological MRI measurements. Brain Behav (2017) 7(9):e00759. doi: 10.1002/brb3.759 28948069 PMC5607538

[41] Nerland S Jorgensen KN Nordhoy W Maximov Ii Bugge RaB Westlye LT . Multisite reproducibility and test-retest reliability of the T1w/T2w-ratio: A comparison of processing methods. Neuroimage (2021) 245:118709. doi: 10.1016/j.neuroimage.2021.118709 34848300

[42] Kong Z Yan C Zhu R Wang J Wang Y Wang Y . Imaging biomarkers guided anti-angiogenic therapy for malignant gliomas. NeuroImage Clin (2018) 20:51–60. doi: 10.1016/j.nicl.2018.07.001 30069427 PMC6067083

[43] Sourbron SP Buckley DL . Classic models for dynamic contrast-enhanced MRI. NMR BioMed (2013) 26(8):1004–27. doi: 10.1002/nbm.2940 23674304

[44] Sourbron SP Buckley DL . On the scope and interpretation of the tofts models for DCE-MRI. Magn Reson Med (2011) 66(3):735–45. doi: 10.1002/mrm.22861 21384424

[45] Li KL Lewis D Coope DJ Roncaroli F Agushi E Pathmanaban ON . The LEGATOS technique: A new tissue-validated dynamic contrast-enhanced MRI method for whole-brain, high-spatial resolution parametric mapping. Magn Reson Med (2021). doi: 10.1002/mrm.28842 33991126

[46] Kwee TC Galban CJ Tsien C Junck L Sundgren PC Ivancevic MK . Comparison of apparent diffusion coefficients and distributed diffusion coefficients in high-grade gliomas. J Magn Reson Imaging (2010) 31(3):531–7. doi: 10.1002/jmri.22070 PMC2918396 20187193

[47] Shukla-Dave A Obuchowski NA Chenevert TL Jambawalikar S Schwartz LH Malyarenko D . Quantitative imaging biomarkers alliance (QIBA) recommendations for improved precision of DWI and DCE-MRI derived biomarkers in multicenter oncology trials. J Magn Reson Imaging (2019) 49(7):e101–e21. 10.1002/jmri.26518 PMC6526078 30451345

[48] OSIPI task force 1.2: DCE/DSC software inventory . Available at: https://osipi.org/task-force-1-2/.

[49] Scouten A Papademetris X Constable RT . Spatial resolution, signal-to-noise ratio, and smoothing in multi-subject functional MRI studies. Neuroimage (2006) 30(3):787–93. doi: 10.1016/j.neuroimage.2005.10.022 16343951

[50] Winfield JM Payne GS Weller A Desouza NM . DCE-MRI, DW-MRI, and MRS in cancer: Challenges and advantages of implementing qualitative and quantitative multi-parametric imaging in the clinic. Top Magn Reson Imaging (2016) 25(5):245–54. doi: 10.1097/RMR.0000000000000103 PMC5081190 27748710

[51] Xue JH Titterington DM . T-tests, f-tests and otsu's methods for image thresholding. IEEE Trans Image Process (2011) 20(8):2392–6. 10.1109/TIP.2011.2114358 21324779

[52] Zhang Y Brady M Smith S . Segmentation of brain MR images through a hidden Markov random field model and the expectation-maximization algorithm. IEEE Trans Med Imaging (2001) 20(1):45–57. doi: 10.1109/42.906424 11293691

[53] Kassambara A . Practical guide to cluster analysis in r: unsupervised machine learning. (2017).

[54] Sammouda R El-Zaart A . An optimized approach for prostate image segmentation using K-means clustering algorithm with elbow method. Comput Intell Neurosci (2021) 2021:4553832. doi: 10.1155/2021/4553832 34819951 PMC8608531

[55] Yamashita R Nishio M Do RKG Togashi K . Convolutional neural networks: An overview and application in radiology. Insights Imaging (2018) 9(4):611–29. doi: 10.1007/s13244-018-0639-9 PMC6108980 29934920

[56] LeCun Y Bengio Y Hinton G . Deep learning. Nature (2015) 521(7553):436–44. doi: 10.1038/nature14539 26017442

[57] Shah GD Kesari S Xu R Batchelor TT O'neill AM Hochberg FH . Comparison of linear and volumetric criteria in assessing tumor response in adult high-grade gliomas. Neuro-oncology (2006) 8(1):38–46. doi: 10.1215/S1522851705000529 16443946 PMC1871928

[58] Menze BH Jakab A Bauer S Kalpathy-Cramer J Farahani K Kirby J . The multimodal brain tumor image segmentation benchmark (BRATS). IEEE Trans Med Imaging (2015) 34(10):1993–2024. doi: 10.1109/TMI.2014.2377694 25494501 PMC4833122

[59] Ottens T Barbieri S Orton MR Klaassen R Van Laarhoven HWM Crezee H . Deep learning DCE-MRI parameter estimation: Application in pancreatic cancer. Med Image Anal (2022) 80:102512. doi: 10.1016/j.media.2022.102512 35709559

[60] Katzendobler S Do A Weller J Dorostkar MM Albert NL Forbrig R . Diagnostic yield and complication rate of stereotactic biopsies in precision medicine of gliomas. Front Neurol (2022) 13:822362. doi: 10.3389/fneur.2022.822362 35432168 PMC9005817

[61] Mook S Bonnefoi H Pruneri G Larsimont D Jaskiewicz J Sabadell MD . Daily clinical practice of fresh tumour tissue freezing and gene expression profiling; logistics pilot study preceding the MINDACT trial. Eur J Cancer (2009) 45(7):1201–8. doi: 10.1016/j.ejca.2009.01.004 19232484

[62] Xing S Freeman CR Jung S Turcotte R Levesque IR . Probabilistic classification of tumour habitats in soft tissue sarcoma. NMR Biomed (2018) 31(11):e4000. doi: 10.1002/nbm.4000 30113738

[63] Bauchet L Mathieu-Daude H Fabbro-Peray P Rigau V Fabbro M Chinot O . Oncological patterns of care and outcome for 952 patients with newly diagnosed glioblastoma in 2004. Neuro-oncology (2010) 12(7):725–35. doi: 10.1093/neuonc/noq030 PMC2940657 20364023

[64] PreOperative brain irradiation in glioblastoma (POBIG) (2022). Available at: https://clinicaltrials.gov/ct2/show/NCT03582514. 10.1016/j.ctro.2023.100585 PMC9947330 36845633

[65] Neoadjuvant chemoradiation for resectable glioblastoma (NeoGlio) (2022). Available at: https://clinicaltrials.gov/ct2/show/NCT04209790.

[66] Jiang H Zeng W Ren X Cui Y Li M Yang K . Super-early initiation of temozolomide prolongs the survival of glioblastoma patients without gross-total resection: A retrospective cohort study. J Neurooncol (2019) 144(1):127–35. doi: 10.1007/s11060-019-03211-1 31175579

[67] Collet S Guillamo JS Berro DH Chakhoyan A Constans JM Lechapt-Zalcman E . Simultaneous mapping of vasculature, hypoxia, and proliferation using dynamic susceptibility contrast MRI, (18)F-FMISO PET, and (18)F-FLT PET in relation to contrast enhancement in newly diagnosed glioblastoma. J Nucl Med (2021) 62(10):1349–56. doi: 10.2967/jnumed.120.249524 PMC8724903 34016725

[68] Chawla S Kavouridis VK Boaro A Korde R Amaral Medeiros S Edrees H . Surgery vs. biopsy in the treatment of butterfly glioblastoma: A systematic review and meta-analysis. Cancers (Basel) (2022) 14(2). doi: 10.3390/cancers14020314 PMC8773472 35053478

[69] Harat M Blok M Miechowicz I Wiatrowska I Makarewicz K Malkowski B . Safety and efficacy of irradiation boost based on 18F-FET-PET in patients with newly diagnosed glioblastoma. Clin Cancer Res (2022) 28(14):3011–20. doi: 10.1158/1078-0432.CCR-22-0171 35552391

[70] Laack NN Pafundi D Anderson SK Kaufmann T Lowe V Hunt C . Initial results of a phase 2 trial of (18)F-DOPA PET-guided dose-escalated radiation therapy for glioblastoma. Int J Radiat Oncol Biol Physics (2021) 110(5):1383–95. doi: 10.1016/j.ijrobp.2021.03.032 33771703

[71] Gondi V Pugh S Tsien C Chenevert T Gilbert M Omuro A . Radiotherapy (RT) dose-intensification (DI) using intensity-modulated RT (IMRT) versus standard-dose (SD) RT with temozolomide (TMZ) in newly diagnosed glioblastoma (GBM): Preliminary results of NRG oncology BN001. Int J Radiat Oncol Biol Physics (2020) 108(3):S22–S3. doi: 10.1016/j.ijrobp.2020.07.2109

[72] Stoyanova R Chinea F Kwon D Reis IM Tschudi Y Parra NA . An automated multiparametric MRI quantitative imaging prostate habitat risk scoring system for defining external beam radiation therapy boost volumes. Int J Radiat Oncol Biol Physics (2018) 102(4):821–9. doi: 10.1016/j.ijrobp.2018.06.003 PMC6245650 29908220

[73] Paldino MJ Barboriak D Desjardins A Friedman HS Vredenburgh JJ . Repeatability of quantitative parameters derived from diffusion tensor imaging in patients with glioblastoma multiforme. J Magn Reson Imaging (2009) 29(5):1199–205. doi: 10.1002/jmri.21732 19388113

[74] Jafari-Khouzani K Emblem KE Kalpathy-Cramer J Bjornerud A Vangel MG Gerstner ER . Repeatability of cerebral perfusion using dynamic susceptibility contrast MRI in glioblastoma patients. Transl Oncol (2015) 8(3):137–46. doi: 10.1016/j.tranon.2015.03.002 PMC4486737 26055170

[75] Jardim-Perassi BV Huang S Dominguez-Viqueira W Poleszczuk J Budzevich MM Abdalah MA . Multiparametric MRI and coregistered histology identify tumor habitats in breast cancer mouse models. Cancer Res (2019) 79(15):3952–64. doi: 10.1158/0008-5472.CAN-19-0213 PMC6677627 31186232

[76] Jardim-Perassi BV Mu W Huang S Tomaszewski MR Poleszczuk J Abdalah MA . Deep-learning and MR images to target hypoxic habitats with evofosfamide in preclinical models of sarcoma. Theranostics (2021) 11(11):5313–29. doi: 10.7150/thno.56595 PMC8039958 33859749

[77] Burger PC Heinz ER Shibata T Kleihues P . Topographic anatomy and CT correlations in the untreated glioblastoma multiforme. J Neurosurg (1988) 68(5):698–704. doi: 10.3171/jns.1988.68.5.0698 2833587

[78] Thomas NWD Sinclair J . Image-guided neurosurgery: History and current clinical applications. J Med Imaging Radiat Sci (2015) 46(3):331–42. doi: 10.1016/j.jmir.2015.06.003 31052141

[79] Huszar IN Pallebage-Gamarallage M Foxley S Tendler BC Leonte A Hiemstra M . Tensor Image registration library: Automated non-linear registration of sparsely sampled histological specimens to post-mortem MRI of the Whole Human Brain. bioRxiv 849570

