# Supplementary figures and images for: Visualising spatial heterogeneity in glioblastoma using imaging habitats
Source: Front Oncol. 2022 Nov 24;12:1037896. doi: 10.3389/fonc.2022.1037896 (PMC9731157; doi:10.3389/fonc.2022.1037896)

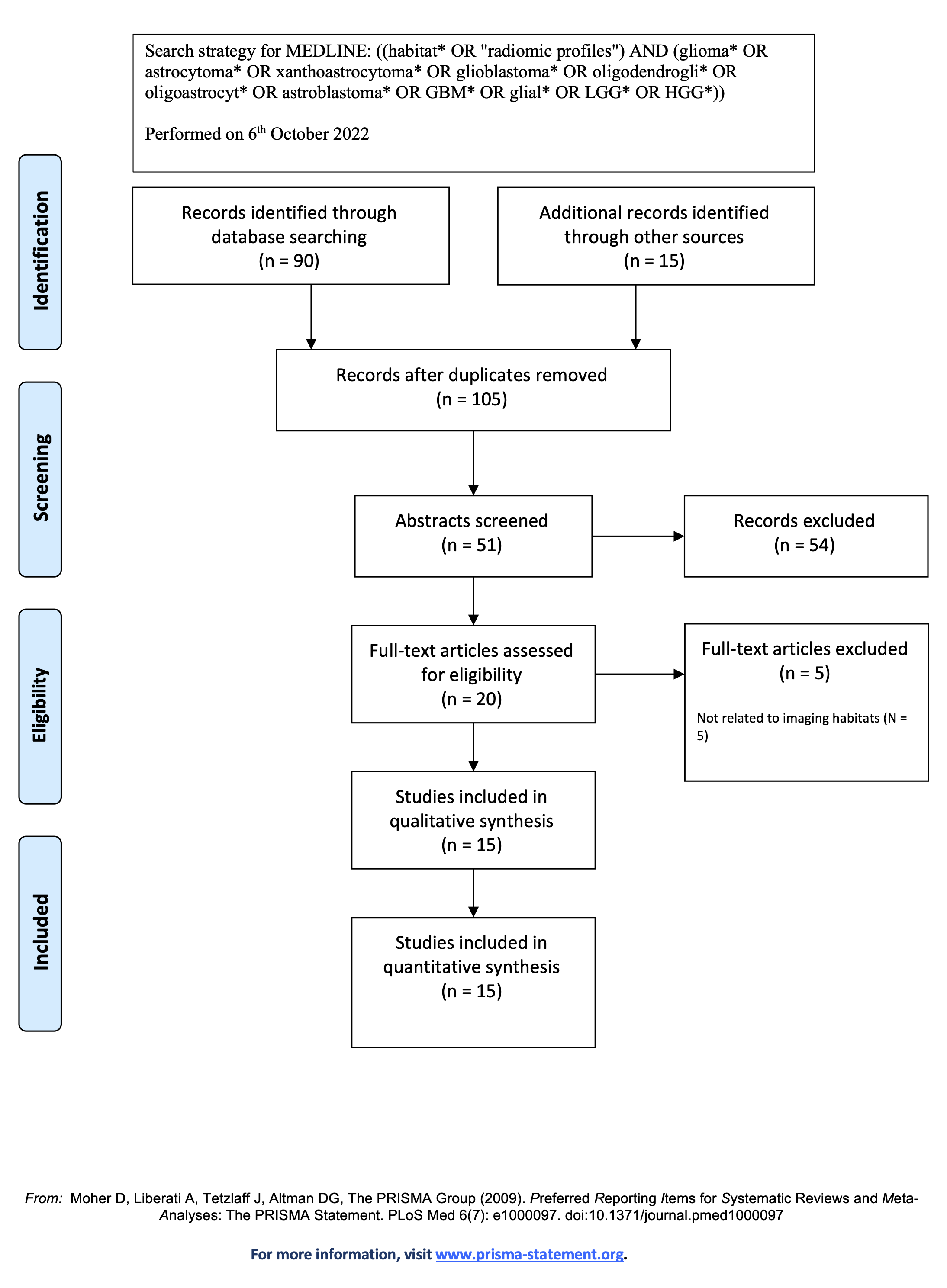

Supplement: Supplementary Figure 1 — Search strategy. To review the current evidence on imaging habitats, MEDLINE was queried systematically using the search terms described by two independent authors (MW, PVH) with discrepancies resolved through discussion with a third (EH). A total of 15 articles were included in the final review relating to imaging habitats that are summarised in . [file Image_1.tiff]
